# Prevalence and factors associated with suboptimal blood pressure among ambulatory patients with diabetic kidney disease attending a tertiary hospital in Uganda

**DOI:** 10.4314/ahs.v23i2.57

**Published:** 2023-06

**Authors:** David Wamala, Edrisa Mutebi, James Kayima

**Affiliations:** Department of Medicine, School of Medicine, College of Health Sciences, Makerere University, P. O. Box 7072, Kampala, Uganda

**Keywords:** Blood pressure, diabetes mellitus, kidney disease

## Abstract

**Background:**

Kidney failure prevalence is increasing among diabetic kidney disease (DKD) patients in low-income countries. Suboptimal blood pressure (BP) drives kidney failure and mortality. The burden of suboptimal BP and its associated factors among these patients are not well documented.

**Objectives:**

To determine the prevalence of suboptimal BP and associated factors among those with DKD attending Kiruddu National Referral Hospital.

**Methods:**

In this cross-sectional study, recruited participants were screened for DKD using urine dipsticks from 6^th^ May 2020 to 15^th^ July 2020. A pretested questionnaire was administered, BP, height and weight were measured. Suboptimal BP was defined as systolic BP > 130mmHg and or diastolic BP > 80mmHg. A Poisson regression model analysed the associated factors.

**Results:**

250 participants with DKD, mean age of 52(11) years were included of whom 199 (79.6%) were female. Suboptimal BP prevalence was 84.4%, associated with past (p = 0.04) and current (p < 0.001) alcohol use, overweight (p < 0.001) and obesity (p < 0.001), wage earning (p < 0.001) and professionals (p = 0.048).

**Conclusion:**

The prevalence of suboptimal BP was high among the overweight, obese, unemployed and alcohol users. Thus, there is a need for better BP control interventions

## Introduction

In 2019, the global prevalence of Diabetes mellitus (DM) was estimated at 9.3% and is expected to rise to 10.2% by 2030 and to 10.9% by 2045^1^. An estimated 19.4 million people aged 20 -79 years in Africa have DM^2^. In Uganda, the prevalence was 1.4% in 2016^3^. Africa has the highest proportion (60%) of people with undiagnosed DM in the world^2^. The prevalence of uncontrolled BP is higher among patients with DM than in the general population and this coexistence increases the incidences of cardiovascular disease (CVD), end-stage kidney disease (ESKD) and mortality^4^. Optimal BP is therefore paramount despite controversies about targets. The American Heart Association (AHA) recommends a target systolic BP < 130 mm Hg and diastolic BP < 80 mm Hg especially among those with increased CVD risk like diabetic kidney disease (DKD)^5^.

Diabetic kidney disease is characterized by the abnormal passage of albumin in urine and or a steady decline in kidney function leading to ESKD^6^. The burden of DKD is rising continuously with disparate growth in low to middle-income countries and it's still under-recognized as a global burden of disease^7^. In Sub-Saharan Africa, the burden of DKD is high ranging from 11% to 83.7%^8^ and in Uganda, it is estimated at 47.4%^9^. End-Stage Kidney Disease, the result of DKD causes economic loss to the affected individuals and their families due to the high costs towards tertiary management of this chronic ailment^7^. With few nephrologists and dialysis centres, the outcome of these patients is dire^10^.

Suboptimal BP among patients with DKD is an independent risk factor for the development of ESKD and CVD in the form of coronary artery disease, left ventricular hypertrophy, valvular heart diseases, stroke and arrhythmias like atrial fibrillation^11^. Therefore, optimal BP among these patients is critical in preventing the progression to CVD, ESKD and mortality^12^. Unfortunately, information on the burden of suboptimal BP and the associated factors among patients with DKD in Sub-Saharan Africa is limited and this could hinder efforts to improve its control in this vulnerable patient population leading to bad outcomes. The aim of the study was therefore to determine the prevalence of suboptimal BP and the associated factors among ambulatory patients with DKD attending a tertiary hospital in Uganda.

## Methods

### Study design

This was a cross-sectional study done among ambulatory participants with DM who attended the DM clinic of Kiruddu National Referral Hospital (KNRH) from 6^th^ May 2020 to 15^th^ July 2020.

### Study setting

Kiruddu National Referral Hospital is a tertiary hospital with a bed capacity of 170 and located in Kampala city. The DM clinic runs once a week with an average attendance of 70 -100 patients. On each clinic day, the patients are first taken through a group health education session by a nursing officer followed by registration, blood sugar and BP check-ups. The patients are then seen by a team which comprises endocrinologists, senior house officers and medical officers. The patients seen in this clinic include new referrals, those on follow up after discharge from inpatient care and those already in the clinic on regular reviews and drug refills.

### Sample size calculation

Sample size for prevalence of suboptimal blood pressure control was calculated Using Kish and Leslie formula (1965): **N = Z^2^ P (1 - P) / D^2^**.

**N** - Sample Size.

**Z** - 1.96, corresponding to the 95% confidence interval.

**P** - 0.863, prevalence of suboptimal blood pressure among Sample predialysis participants including those with DM 13

**D** - 0.05, the accepted absolute error.

Therefore, N = 1.962 X 0.863 (1 - 0.863) / 0.052 = 182 participants.

Sample size for factors associated was calculated using the formula


$${\text{n}}_{\text{per group }}=\frac{{\left({\text{z}}_{\alpha /2}+{\text{z}}_{1-\beta }\right)}^{2}*\left[\left({\text{P}}_{1}*\left(1-{\text{P}}_{1}\right)\right)+\left({\text{P}}_{0}*\left(1-{\text{P}}_{0}\right)\right)\right]}{{\left[{\text{P}}_{1}-{\text{P}}_{0}\right]}^{2}}$$

**n -** Number of participants per group

**Zα/2 =** 1.96, corresponding to a 5% error.

**Z1 - β =** 0.84 corresponding to power of 80%.

**P1 =** 0.539, the prevalence of suboptimal blood pressure among participants with DM who were employed 14

**P0 =** 0.333, the prevalence of suboptimal blood pressure among participants with DM who were overweight 14

Therefore, n per group = (1.96 + 0.84)^2^ ((0.539 X (1 - 0.539) + (0.333 X (1 - 0.333)) / (0.539 -0.333)^2^ = 250 participants. Therefore, the required sample size for the study was 250 participants.

### Eligibility and subject recruitment

Adults with DM aged 18 years or older who had given written informed consent were consecutively recruited and had their urine screened for albumin. Only those with albuminuria on urine dipstick in the absence of leukocyte esterase were included in the study.

### Procedures

The study was conducted among patients with DM presenting to the study site. Diabetic kidney disease was diagnosed through the assessment of urine albumin. Eligible participants were asked to collect 50mls of urine in a sterile container using a clean catch method. The urine was screened for albumin using a dipstick. Only those with albumin on urine dipstick and negative leucocyte esterase were included in the study. Two BP readings were taken 5 minutes apart using a calibrated Zayo sigma BSP-11 model BP monitor. The average of these two readings was calculated and recorded. The weight (kilograms) and height (meters) of these patients were measured using a calibrated Seca automated weighing scale model 876 and a calibrated, fixed stationary height meter respectively. The body mass index (BMI) was calculated and a pretested questionnaire administered.

### Study definitions

Diabetes mellitus: Any patient aged 18 years and above with a previously documented diagnosis of DM based on either a fasting blood sugar ≥ 7mmol/l or oral glucose tolerance test ≥ 11.1 mmol/l or HbAlc ≥ 6.5%^2^.

Suboptimal BP: Systolic BP > 130 mm Hg and or diastolic BP > 80 mm Hg according to AHA^5^

Diabetic kidney disease: Presence of albuminuria with negative leukocyte esterase on urine dipstick among patients with DM^6^.

The BMI (kg / m^2^) was classified as: Underweight < 18.5, Normal (18.5 - 24.9), Overweight (25 - 29.9), obese ≥ 30^13^.

### Data management

The collected data were checked, organized and coded before double entry. The data were entered using EPI-DA-TA, cleaned, validated and exported to STATA version 14 for analysis.

### Data Analysis

The prevalence was determined using the percentage of patients with suboptimal BP with its 95% confidence interval. The factors associated with suboptimal BP were determined using the Poisson regression model with robust standard errors. Bivariate analysis was performed for each of the independent variables to determine factors independently associated with suboptimal BP using odds ratios (OR) and p-values. Factors with p ≤ 0.2 were considered for multivar independent variables with p ≤ 0.05 were identified using the stepwise backward method and interaction was assessed by comparing the -2 Log likelihoods of the reduced and full models. Confounding was assessed basing on a difference of ≥ 10% between the crude and adjusted OR of the variables.

### Ethical consideration

Ethical approval was sought from the school of medicine and KNRH research and ethics committees. Written informed consent was obtained from all study participants after a full explanation of the objectives and procedures of the study. Those with significant abnormal urine and BP results were treated accordingly.

## Results

**Figure 1:**
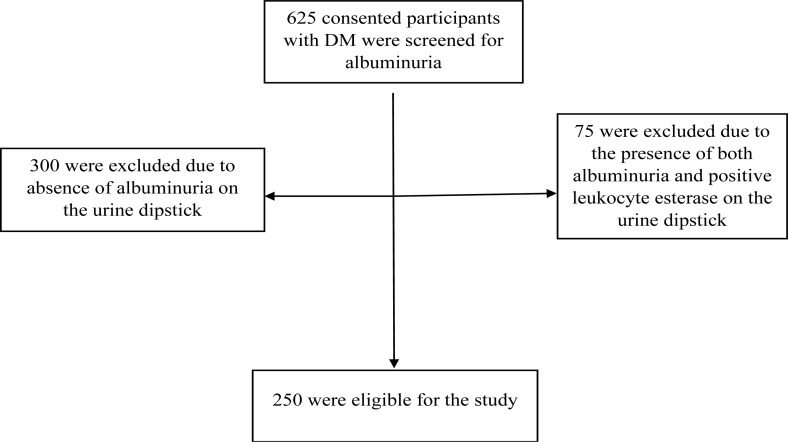
Study recruitment diagram of ambulatory participants with DKD who attended the DM clinic of KNRH

The study recruitment process is shown in figure 1 below.

Tables 1 and 2 below show the socio-demographic and clinical characteristics respectively of the study participants. The majority, 190 (76.0%) of the participants were in the age range of 31 - 60 years and 199 (79.6%) of all the study participants were female.

**Table 1:** Socio-demographic characteristics of ambulatory participants with DKD who attended the DM clinic of KNRH

Characteristic	Frequency	Percentage
**Age in years,** mean (SD), 52 (11)		
**Sex**		
Male	51	20.4
Female	199	79 .6
**Education level**		
None	33	13.2
Primary	125	50.0
Secondary	76	30.4
Tertiary	16	6.4
**Occupation**		
Unemployed	183	73.2
Wage earner	59	23.6
Professional	8	3.2
**History of smoking**		
Never	221	88.4
Past smoker (stopped ≥ 6 months ago)	21	8.4
Current smoker	8	3.2
**Alcohol use**		
Never	43	17.2
Past alcohol user (≥ 6 months ago)	26	10.4
Current alcohol user	181	72.4
**Place of residence**		
Rural	48	19.2
Urban	202	80.8

**Table 2:** Clinical characteristics of ambulatory participants with DKD who attended the DM clinic of KNRH

Characteristics	Frequency	Percentage
**Duration in years since DM diagnosis median (IQR)** **Duration in years since DM diagnosis**	8(4, 13)	
≤ 10	168	67.2
11 - 20	64	25.6
≥ 21	18	7.2
**History of hypertension**		
Yes	184	73.6
No	66	26.4
**Duration in years since hypertension diagnosis median (IQR)** **Duration in years since hypertension diagnosis**	8(4, 14)	
≤ 10	125	67.93
11 - 30	53	28.8
≥ 31	6	3.26
**Statin use**		
Yes	44	17.60
No	206	82.40
**Number of glucose-lowering drugs used**		
1 drug	71	28.4
2 drugs	170	68.0
≥ 3 drugs	9	3.60
**Number of antihypertensive drugs used**		
1 drug	33	17.9
2 drugs	62	33.7
≥ 3 drugs	89	48.4
**Body mass index (kg/m^2^)**		
18.5 - 24.9	56	22.40
25 - 29.9	148	59.20
≥ 30	46	18.40

### Prevalence of suboptimal BP among ambulatory participants with DKD

**Figure 2:**
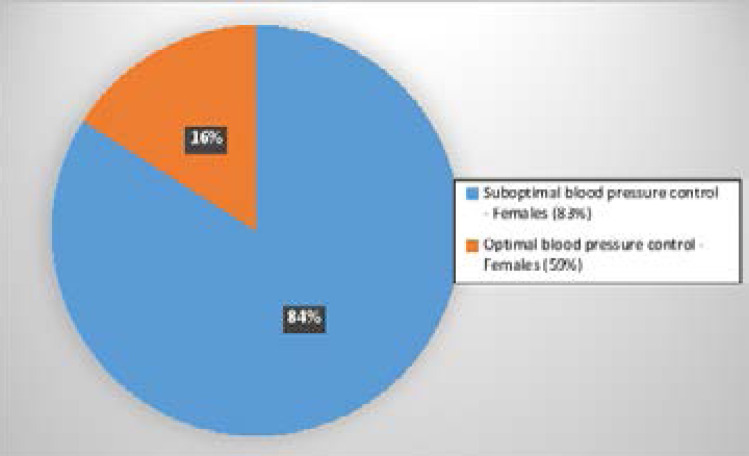
Prevalence of suboptimal BP among ambulatory participants with DKD

Of the 250 participants recruited in the study, 211 (84.4%) 95% CI: 79.3 - 88.4 had suboptimal BP as shown in figure 2 below. Among females, 176 (88.4%) had suboptimal BP compared to 35 (68.6%) of the males.

The majority161 (84.7%) of the participants with suboptimal BP were in the age category of 31 - 60 years, 111 (88.8%) had primary education as the highest academic level, 189 (85.5%) had never smoked and 170 (84.2%) resided in urban areas.

### Factors associated with suboptimal blood pressure control among participants with DKD

At bivariate analysis, the factors associated with suboptimal blood pressure control were sex, occupation, alcohol use, family history of DM, history of hypertension, BMI and using statins as shown in table 3 below.

**Table 3:** Multivariate analysis of factors associated with suboptimal BP among ambulatory participants with DKD

Characteristic	Sub-optimal BP	Optimal BP	aOR	95%CI	P-value
**Occupation**					
Unemployed	179(97.81)	4(2.19)	1		
Wage earner	30(50.85)	29(49.15)	0.68	0.58, 0.81	**<0.001**
Professional	2(25.00)	6(75.00)	0.37	0.14, 0.99	**0.048**
**Alcohol use**					
Never	18(41.86)	25(58.14)	1		
Past alcohol user	17(65.38)	9(34.62)	1.37	1.01, 1.85	**0.040**
(≥ 6 months ago)					
Current alcohol user	176(97.24)	5(2.76)	1.57	1.23, 2.00	**<0.001**
**Body mass index (kg/m^2^)**					
18.5 - 24.9	22(39.29)	34(60.71)	1		
25 - 29.9	144(97.30)	4(2.70)	1.82	1.40, 2.38	**<0.001**
≥ 30	45(97.83)	1(2.17)	1.89	1.44, 2.47	**<0.001**

Multivariate analysis showed occupation, alcohol use and body mass index to be associated with suboptimal blood pressure control as shown in Table 4 below. Participants who were wage earners (OR = 0.68, 95% CI: 0.58 - 0.81, p < 0.001) or professionals (OR = 0.37, 95% CI: 0.14 - 0.99, p = 0.048) had lower odds of suboptimal blood pressure control as compared to those who were unemployed. The odds of suboptimal blood pressure control among participants with past (OR = 1.37, 95% CI: 1.01 - 1.85, p = 0.04) or current (OR = 1.57, 95% CI: 1.23 - 2, p < 0.001) alcohol use were 1.4 and 1.6 times respectively the odds of those without a history of alcohol use. The odds of suboptimal blood pressure control among participants who were overweight (OR = 1.82, 95% CI: 1.4 - 2.38, p < 0.001) or obese (OR = 1.89, 95% CI: 1.44 - 2.47, p < 0.001) were 1.8 and 1.9 times respectively the odds of those with normal weight.

**Table 4:** Multivariate analysis of factors associated with suboptimal blood pressure control among ambulatory participants with DKD

Characteristic	Sub-optimal blood pressure control	Optimal blood pressure control	aOR	95%CI	P-value
**Occupation**					
Unemployed	179(97.81)	4(2.19)	1		
Wage earner	30(50.85)	29(49.15)	0.68	0.58, 0.81	**<0.001**
Professional	2(25.00)	6(75.00)	0.37	0.14, 0.99	**0.048**
**Alcohol use**					
Never	18(41.86)	25(58.14)	1		
Past alcohol user	17(65.38)	9(34.62)	1.37	1.01, 1.85	**0.040**
(≥ 6 months ago)					
Current alcohol user	176(97.24)	5(2.76)	1.57	1.23, 2.00	**<0.001**
**Body mass**					
**index (kg/m^2^)**					
18.5 - 24.9	22(39.29)	34(60.71)	1		
25 - 29.9	144(97.30)	4(2.70)	1.82	1.40, 2.38	**<0.001**
≥ 30	45(97.83)	1(2.17)	1.89	1.44, 2.47	**<0.001**

## Discussion

We found the prevalence of suboptimal BP among ambulatory participants with DKD as high as eight in ten. This could be because we used a lower BP cut off of 130/80mmHg thus identifying more participants. White coat hypertension could also have affected the BP measurements as they were measured in a clinic environment. The majority of these patients could have had comorbid kidney dysfunction and hence high BP but this could not be confirmed since the serum creatinine and glomerular filtration rate were not determined. Other possible explanations could be poor medication adherence, poor appointment keeping among others but these were not assessed in this study. This high prevalence of suboptimal BP implies that the majority of these patients are at increased risk of CVD, ESKD and mortality. Our findings are consistent with other African studies that used similar BP cut offs^14^,^15^. This similarity is because of similar settings with the same health system, health care professional and patient-related barriers to optimal BP control. Health system-related barriers include poor universal health insurance coverage leading to out-of-pocket payment by most patients which affects access and adherence to treatment^16^. There's a lack of policies on BP medication procurement and distribution resulting in frequent stock shortages^16^. There's poor implementation of BP control guidelines, clinician inertia and a low physician to patient ratio among others^16^. Patients have poor awareness about BP control, poor adherence to medications and are reluctant to change life styles^16^. Surprisingly, a study in a high-income country (France) also found a similarly high prevalence of suboptimal BP among ambulatory patients with DKD majorly explained by poor medication adherence among the study participants^17^.

We found a significant positive association between sub-optimal BP with current (p = 0.04) and past (p < 0.001) alcohol use. This could be explained by the fact that alcohol use interferes with medication adherence because it causes financial constraints which affect the acquisition of the necessary BP lowering medications. It could also interact with BP lowering medications hence affecting their actions or increasing their side effect profile. The reasons why the majority of participants were taking alcohol, as well as the quantity of alcohol taken, were not ascertained in this study. Our findings are consistent with other studies done in South Africa and Uganda^18^,^19^. Biologically, alcohol causes an imbalance between central nervous system factors influencing cardiac output and peripheral resistance, enhances sympathetic activity, stimulates the renin-angiotensin-aldosterone system and increases cortisol levels among other actions^20^.

We found a significant positive association between suboptimal BP among participants who were overweight (p < 0.001) and obese (p < 0.001). This could be explained by the low level of physical activity associated with being overweight or obese leading to blood pressure raise. The level of physical activity among the participants however was not assessed in this study. This implies that the majority of participants who were overweight or obese have an increased risk of CVD, ESKD and mortality. Our findings are consistent with findings from several other African studies^21^,^22^,^23^. The biological explanations for this association include; The excess fat accumulation in and around the kidneys in overweight and obese individuals is associated with increased intrarenal pressures, impaired pressure mediated loss of urinary sodium leading to increased blood volume and hence elevated BP^24^. Secondly, obese and overweight individuals, especially those with visceral fat accumulation often have mild to moderate increases in plasma renin activity, increased angiotensinogen blood levels, increased angiotensin-converting enzyme activity and hence increased angiotensin II and aldosterone^25^. Thirdly, obesity generally decreases parasympathetic tone and increases sympathetic nervous activity. These changes in autonomic activity are associated with increased heart rate, decreased heart rate variability and reduced baroreflex sensitivity leading to elevated BP^26^.

We found that being a wage earner (p < 0.001) or a professional (p = 0.048) were protective against sub-optimal BP as compared to being unemployed. This could be because having an income enables patients to buy the necessary medications that may not be stocked in public hospitals and also keep clinic appointments. Of note, the majority of participants with suboptimal BP were unemployed and this implies increased risks of CVD, ESKD and mortality. Thus, there's a need to have a continuous supply of the basic BP lowering medications to control BP among these patients and improve outcomes. These findings are consistent with several other African studies 18,27,28,29. Being unemployed is linked to high poverty levels in Sub-Saharan Africa which hinders the ability to buy the necessary medications which sometimes are not stocked in government hospitals and also makes it difficult for patients to keep clinic appointments due to the high out of the pocket expenditure. This makes monitoring and treatment of suboptimal BP very difficult and thus increases the risk of progression to ESKD, CVD and eventual mortality^7^. This situation is complicated by the fact that this group is unlikely to afford advanced therapies for ESKD^6^.

## Strength and limitations of the study

Our study assessed suboptimal BP in a high-risk patient population which few studies have done before. Our limitations were using clinic BP measurements that are subject to the white coat effect and white coat hypertension phenomena. We tried to minimize this by taking two BP measurements at least 5 minutes apart. We couldn't solely attribute the albuminuria to DM since we did not do kidney biopsies. In our study, we did not assess aspects of medication adherence, retention in care, appointment keeping and patient and provider perspectives on suboptimal BP.

## Conclusion

We found a high prevalence of suboptimal BP among patients with DKD in our setting especially among those who are overweight, obese, unemployed and alcohol users. This implies an increased risk of CVD, ESKD and mortality. Thus, we recommend routine screening for albuminuria among patients with DM to properly identify, treat and follow up those who need stringent BP control for better outcomes. Further research is needed to assess aspects of medication adherence, retention in care, appointment keeping and patient and provider perspectives on suboptimal BP.
